# The Summer Bowel Affections of Children

**Published:** 1874-08-15

**Authors:** 


### THE SUMMER BOWEL AFFECTIONS OF CHILDREN.

AT the last meeting of the Chicago Society of Physicians and Surgeons, a very interesting discussion was held regarding the summer bowel affections of children, a full report of which appears in the proceedings of the Society as given in the present number of the EXAMINER. The subject is especially *apropos* at this season, when this class of affections is so prevalent in all our larger cities.

In a late correspondence received from Dr. B. S. Woodworth, of Fort Wayne, Ind., he states his belief in the essentially malarious origin of cholera infantum and the kindred bowel affections of children. Quinine in combination with opiates he has found most efficient in controlling these cases. He usually combines them as in the following formula:

℞.—Quiniæ sulph., grs. xxv.
Tannin, grs. x.
Tinct. opii, gtts. xx.
Ess. menth. pipt., gtts. xx.
Syr. simpl., ℥ ii.

M. From half a teaspoonful to a teaspoonful, according to age, to be given every two hours until vomiting and purging ceases.

Dr. Woodworth has had a large experience in the observation and treatment of children's diseases for the past twenty-five years, and his evidence, given as the result of long experience, is therefore of especial value.

These bowel affections of children and the accompanying symptoms which they occasion, undoubtedly vary, however, materially in the type and character which they assume in different localities and in different seasons in the same locality. In the eastern and sea-board cities the malarial element will be found much less evident and frequently manifest than in our southern and western cities. When the distinct exacerbations of fever, and the generally intermitting character of all the phenomena indicate the presence of a malarial element in the disease, quinine is, of course, indicated. In cases of cholera infantum, however, when vomiting and purging is at all active, we have scarcely ever been able to administer the quinine in any form that would be retained upon the stomach. We more frequently, therefore, substitute for it, in such cases, small doses of phloridzine combined as in the following formula:

℞.—Phloridzinæ, grs. xxiv.
Spts. ammon. arom., ʒ i.
Tinct. opii camph., ℥ i.
Syr. simpl., ℥ ss.
Aquæ, ℥ iss.
M. Dose for a child one year old, half a teaspoonful repeated every two or three hours.

This forms a mixture rather agreeable to the taste and acceptable to the stomach, while combining a diffusible stimulant with the anti-periodic and anodyne influences.

F. H. D.

OAT-MEAL FARINA AS A FOOD FOR INFANTS.—MM. Beaumetz and Hardy recommend very highly the use of oat-meal farina in the feeding of young children. According to these gentlemen, this farina resembles human milk most closely in its plastic and respiratory elements, and contains, in addition, iron and phosphate of lime. It has, besides, the property of preventing or arresting the diarrhœa which so frequently occurs in young children. Some infants of four to eleven months, who were fed upon this farina, were found to grow equally well with those who were nourished by the milk of a good nurse.

